# Bluetongue in ruminants: Global epidemiology, pathogenesis, and advances in diagnostic and control strategies within a One Health framework

**DOI:** 10.14202/vetworld.2025.3070-3093

**Published:** 2025-10-20

**Authors:** Siti Rani Ayuti, Aswin Rafif Khairullah, Mirni Lamid, Sunaryo Hadi Warsito, Mohammad Anam Al Arif, Eun Joong Kim, Ikechukwu Benjamin Moses, Sangsu Shin, Bantari Wisynu Kusuma Wardhani, Wasito Wasito, Andi Thafida Khalisa, Riza Zainuddin Ahmad

**Affiliations:** 1 Doctoral Program of Veterinary Science, Faculty of Veterinary Medicine, Universitas Airlangga, Surabaya, East Java, Indonesia; 2 Laboratory of Biochemistry, Faculty of Veterinary Medicine, Universitas Syiah Kuala, Banda Aceh, Indonesia; 3 Research Center for Veterinary Science, National Research and Innovation Agency (BRIN), Bogor, West Java, Indonesia; 4 Department of Animal Husbandry, Faculty of Veterinary Medicine, Universitas Airlangga, Surabaya, East Java, Indonesia; 5 Department of Animal Science and Biotechnology, Kyungpook National University, Sangju, Republic of Korea; 6 Department of Applied Microbiology, Faculty of Science, Ebonyi State University, Abakaliki, Nigeria; 7 Research Center for Pharmaceutical Ingredients and Traditional Medicine, Bogor, West Java, Indonesia; 8 The Pharmacist Professional Education Program, Faculty of Military Pharmacy, Universitas Pertahanan, Bogor, West Java, Indonesia

**Keywords:** bluetongue virus, *Culicoides* vectors, diagnostics, epidemiology, livestock disease control, One Health, vaccination

## Abstract

Bluetongue (BT) is an economically important viral disease of domestic and wild ruminants, caused by the bluetongue virus (BTV), and transmitted primarily by Culicoides midges. The virus has at least 28 known serotypes and several emerging strains, with its distribution expanding beyond traditional endemic zones due to climate change and global trade. This review summarizes recent developments in the epidemiology, molecular characterization, diagnostics, vaccines, and control of BT, with an emphasis on its implications within the One Health framework. A comprehensive literature search covering studies from 2000 to 2025 revealed significant outbreaks in Europe (2024–2025) involving BTV-3 and BTV-12, as well as the emergence of novel serotypes in Asia and Africa. Global economic losses exceed USD 3 billion annually due to mortality, production losses, and trade restrictions. Advances in molecular diagnostics, such as reverse transcription polymerase chain reaction, whole-genome sequencing, and rapid field assays like loop-mediated isothermal amplification and clustered regularly interspaced short palindromic repeats -based platforms, have improved surveillance and serotype identification. Although vaccination remains the cornerstone of BT control, current live and inactivated vaccines are limited by serotype specificity and reassortment risks, highlighting the need for new-generation virus-like particle, recombinant, DNA, and mRNA-based vaccines. Persistent challenges include the absence of differentiating infected from vaccinated animals -compatible polyvalent vaccines, incomplete knowledge of wildlife reservoirs, and uneven surveillance capacities worldwide. Strengthening integrated vector management, genomic monitoring, and climate-informed control strategies through a coordinated One Health approach will be vital to reduce the global burden of bluetongue.

## INTRODUCTION

Bluetongue (BT) is an infectious, non-contagious viral disease transmitted by *Culicoides* biting midges [1]. It is caused by the BT virus (BTV), a member of the family *Reoviridae* and genus *Orbivirus* [2]. The disease affects a wide range of domestic and wild ruminants, including sheep, goats, cattle, camels, llamas, antelopes, and deer. Among these, sheep are the most clinically susceptible, whereas cattle generally exhibit milder or subclinical infections [1]. Due to its substantial economic and animal health impacts, the World Organization for Animal Health (WOAH) classifies BT as a notifiable disease of global concern [3]. Importantly, BTV is not zoonotic and does not infect humans.

Historically, BT was first described in Cape Town, South Africa, in the late eighteenth century, from where it spread to other African nations and subsequently to Europe, Asia, and the Americas [4]. To date, BTV has been detected on every continent except Antarctica. The disease derives its name from one of its hallmark signs, cyanosis of the tongue (“blue tongue”), resulting from vascular thrombosis, edema, and hemorrhage caused by viral damage to endothelial cells [5]. Clinical manifestations include high fever, inflammation of the tongue and intestinal mucosa, lameness due to sore hooves, and necrotic foci in the nasal and buccal mucosa [6].

Accurate diagnosis of BT is vital for disease control and for maintaining safe international trade in animals and animal products [7]. Tentative diagnosis may rely on clinical signs and gross lesions, but confirmation requires laboratory testing. Standard methods include detection of viral RNA by reverse transcription polymerase chain reaction (RT-PCR) and virus isolation in mammalian or insect cell cultures or embryonated chicken eggs [8]. Molecular typing and characterization provide precise identification, while group-specific antigen capture enzyme-linked immunosorbent assay (ELISA) is commonly used to confirm viral isolates [9].

Globally, BT imposes considerable economic losses estimated at approximately USD 3 billion annually [10]. Direct losses include high morbidity and mortality, abortions, stillbirths, low birth weight, reduced fertility, decreased milk yield, and premature culling [11]. Indirect losses stem from restrictions on trade in live ruminants, animal products, and germplasm, as well as costs associated with vaccination, diagnostics, vector control, and supportive treatment of affected animals [11].

The socioeconomic consequences of BT are profound, as the disease reduces livestock productivity and disrupts international trade [12]. Its distribution has shifted significantly in recent years, influenced by climate change, which has expanded the range and activity of *Culicoides* vectors [13]. Given its impact on small ruminant health and production, BT is recognized as a major global threat. If its spread continues unchecked, the availability of meat, milk, and other animal products in consumer markets could be severely compromised [12].

Despite decades of research on BTV, several critical gaps remain in understanding and controlling this transboundary disease. Most available vaccines are serotype-specific and offer limited cross-protection, creating challenges in regions where multiple serotypes co-circulate or novel variants emerge. Differentiating Infected from Vaccinated Animals (DIVA)-compatible vaccines, which are essential for surveillance and trade, remain underdeveloped. While molecular tools such as RT-PCR and whole-genome sequencing have advanced detection, their implementation is uneven across endemic regions, particularly in resource-limited settings. The role of wildlife reservoirs and their contribution to long-term viral persistence is poorly characterized, limiting accurate risk assessments at the livestock–wildlife interface. Moreover, climate-driven expansion of *Culicoides* vectors has reshaped global epidemiology, yet predictive modeling of vector ecology remains incomplete, especially in Asia, Africa, and South America. Socioeconomic analyses are also disproportionately skewed toward outbreaks in developed countries, with limited data on the long-term impacts in low- and middle-income nations where small ruminants are a cornerstone of rural livelihoods. These knowledge and implementation gaps constrain the effectiveness of current surveillance, prevention, and control strategies, underscoring the need for a holistic reassessment of BTV under the One Health framework.

This review aims to provide a comprehensive synthesis of current knowledge on the global epidemiology, pathogenesis, and control strategies of BTV, integrating updated evidence from recent outbreaks (2024–2025) in Europe, Asia, and Africa. Specifically, it seeks to (i) summarize advances in diagnostic technologies, including molecular and field-deployable assays; (ii) critically assess the status and limitations of existing vaccines, while highlighting progress in novel platforms such as virus-like particles (VLPs), recombinant proteins, DNA, and messenger RNA (mRNA) vaccines; (iii) evaluate the evolving role of climate change, vector distribution, and wildlife reservoirs in shaping disease dynamics; and (iv) examine the economic and policy implications of BTV, with particular emphasis on trade and food security. By addressing these dimensions, the review provides updated insights for veterinary authorities, researchers, and policymakers, while identifying priority areas for future research. Ultimately, it underscores the importance of integrated, cross-disciplinary, and One Health-based approaches to mitigate the ongoing and emerging threats of BT at global and regional levels.

## REVIEW METHODOLOGY

### Search strategy

A structured literature search was conducted in PubMed, Scopus, Web of Science, and Google Scholar covering the period from January 2000 to May 2025. The search terms included “*Bluetongue virus*,” “*Culicoides* *vectors*,” “*BTV vaccines*,” “*molecular epidemiology*,” “*diagnostics*,” and “*control strategies*.” These keywords were combined using Boolean operators (“AND,” “OR”) to refine the results.

### Eligibility criteria

Studies were included if they met the following conditions: (i) Peer-reviewed publications in English, (ii) research on BTV affecting ruminants, (iii) epidemiological, molecular, clinical, diagnostic, or vaccine-related studies, and (iv) relevant gray literature such as WOAH, United States Department of Agriculture, and European Union reports. Exclusion criteria comprised non-peer-reviewed sources (unless official reports), duplicate records, incomplete data, and articles not written in English.

### Data extraction and organization

All relevant articles were screened and categorized into six thematic areas: (1) Epidemiology and distribution, (2) molecular epidemiology and genomics, (3) clinical features and pathology, (4) diagnostics and surveillance, (5) vaccines and control measures, and (6) socioeconomic and policy aspects. In instances of overlapping or contradictory findings, preference was given to the most recent, comprehensive, or widely cited studies.

### Quality assessment

Although a formal risk-of-bias scoring system was not applied, the methodological rigor of included studies was carefully considered. Priority was given to peer-reviewed original research, large-scale epidemiological surveys, and experimental trials. Key factors assessed included sample size, study design, and clarity in reporting outcomes.

### Data synthesis

The collected evidence was synthesized in a semi-systematic narrative format. Tables and figures were constructed to summarize global epidemiological trends, diagnostic tools, vaccine developments, and control strategies. This approach enabled the integration of diverse evidence sources, identification of consensus areas, and recognition of persisting research gaps.

## ETIOLOGY

BTV, the causative agent of BT, is an insect-borne pathogen that primarily affects ruminants. BTV belongs to the family *Reoviridae* and genus *Orbivirus*, which also includes closely related viruses such as epizootic hemorrhagic disease virus (EHDV) and African horse sickness virus (AHSV) [14].

The virion is non-enveloped, with a three-layered icosahedral protein capsid approximately 90 nm in diameter [2]. Its genome consists of 10 linear double-stranded RNA segments that encode 7 structural and 4 non-structural proteins (NSPs) [15]. The outer capsid is composed of viral protein 2 (VP2) and VP5. VP2, the primary determinant of serotype, exhibits high variability across geographical isolates and is responsible for hemagglutination, receptor binding, and the induction of serotype-specific neutralizing antibodies [16]. VP5, although more conserved, exhibits variations linked to geographical origin and facilitates membrane penetration, thereby facilitating the release of viral particles from endosomes into the cytoplasm [17].

The middle capsid layer comprises VP7 and VP3, hydrophobic proteins that maintain the integrity of the viral core [15]. VP7 is also implicated in insect cell attachment in the absence of VP2 or VP5 [18]. Together, the VP3/VP7 complex protects the viral dsRNA genome during intracellular transport and prevents premature activation of interferon responses [19].

The inner core is primarily formed by VP3, with enzymatic proteins localized at the five-fold symmetry axis. These include the RNA-dependent RNA polymerase VP1, the RNA-capping enzyme VP4, and the helicase VP6 [20]. VP1 enables efficient replication in both insect and mammalian cells, while VP6 exhibits RNA-dependent ATPase and helicase activities [21].

BTV also encodes several NSPs. NS1 contributes to viral cytopathogenesis [22, 23], NS2 facilitates replication and viral core assembly within inclusion bodies [24], and NS3/NS3A function as viroporins that permeabilize membranes to enable virus release, often through budding mechanisms similar to retroviruses [25]. A recently discovered protein, NS4, has been implicated in viral-host interactions [26].

**Figure 1:**
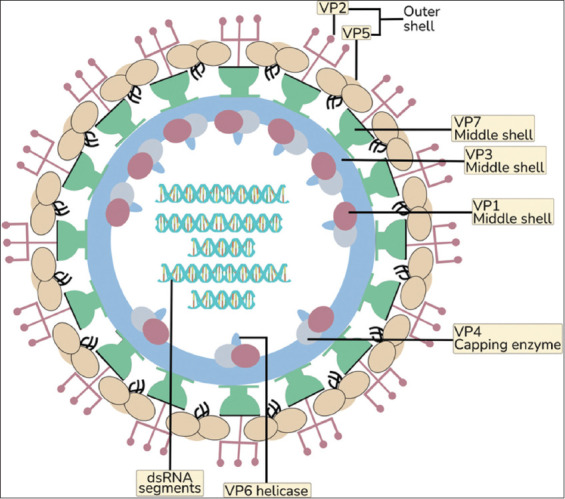
Bluetongue virus protein structure and double-stranded RNA segment [Source: Figure prepared by Bantari Wisynu Kusuma Wardhani].

Figure 1 illustrates the structural organization of BTV, highlighting the capsid proteins and dsRNA genome segments.

## SEROTYPE

Virus isolation and serological analyses have confirmed the existence of 28 classical BTV serotypes worldwide, all of which are capable of inducing BT in ruminants [1]. Serotype determination is primarily based on sequence variations in genome segment 2 (Seg-2), encoding VP2, and to a lesser extent, segment 6 (Seg-6), encoding VP5 [27]. VP2 is the major determinant of antigenicity, responsible for receptor binding and induction of neutralizing antibodies, while VP5 contributes additional variability.

Beyond these classical serotypes, atypical serotypes BTV-25, BTV-26, and BTV-27 have been reported. These are generally non-pathogenic, transmitted by direct contact rather than *Culicoides* midges (similar to BTV-28), and are non-culturable in *Culicoides* cell lines. They have been detected exclusively in small ruminants [28]. For instance, BTV-25 was first reported in a clinically healthy goat in Toggenburg, Switzerland (2007) [29], BTV-26 was isolated from sheep in Kuwait [30], and BTV-27 was detected in goats in Corsica, France (2014) without clinical signs [31].

BTV-28 was identified in the Middle East from contaminated sheeppox and tuberculosis vaccine batches [32]. Unlike atypical strains, BTV-28 induces clinical illness and spreads through direct contact. Phylogenetic analysis of Seg-2 revealed its relatedness to BTV-4, -10, -11, -17, -20, and -24, while Seg-5 closely resembled that of the South African BTV-4 strain and other segments aligned with BTV-26 [32]. Experimental infections in ewes demonstrated the classical clinical manifestations of BT [33].

Recently, three novel putative serotypes have been described. The first, isolated from a South African alpaca, showed close resemblance to BTV-15 based on phylogenetic and cross-neutralization analyses [34]. The second, provisionally named BTV-X ITL2015, was detected in healthy goats in Sardinia, Italy, but has not yet been successfully cultured [35]. Its Seg-2 closely resembled the Chinese isolate BTV-XJ1407 and BTV-27 from Corsica. The third, BTV-XJ1407, was isolated from goats and sheep in China [36].

Globally, serotype distribution is strongly influenced by the presence of competent *Culicoides* species [37]. Genetic analysis shows up to 31.6% nucleotide and 27.4% amino acid variation within strains of the same serotype [38]. Conversely, inter-serotype similarity remains relatively high, with 26.8% nucleotide and 22.2% amino acid identity, complicating classification [39]. Such genomic variability contributes to significant phenotypic and genotypic diversity even within a single serotype [6]. These variants, known as “topotypes,” are geographically associated, forming eastern (Asia, Middle East, Australia, and Mediterranean) and western (Africa, Americas, and Caribbean) lineages [1]. This separation suggests long-term regional evolution with minimal genetic exchange and only sporadic point mutations [40].

Unlike many RNA viruses, BTV lacks a proof-reading polymerase, making it highly error-prone during replication [41]. Variability arises from random mutations and segment reassortment [42], facilitating reintroduction of strains into endemic zones or emergence of novel lineages with altered virulence or expanded host/vector ranges. Continuous genomic surveillance and serotype screening are therefore essential for epidemiology, outbreak preparedness, and vaccine matching [4]. Table 1 summarizes classical (BTV-1 to BTV-28), atypical (BTV-25 to BTV-27), and emerging serotypes (e.g., BTV-28 and BTV-XJ1407) [1, 27–33, 35, 36].

**Table 1:** BTV serotypes and main characteristics.

Serotype	Year	Location	Host	Key characteristics/Notes	References
BTV-1 to BTV-28	Various	Worldwide	Ruminants	Classic; causes BT; serotype is determined by Seg-2/VP2 and Seg-6/VP5	[1, 27]
BTV-25	2007	Toggenburg, Switzerland	Goat	Atypical; non-pathogenic, subclinical, transmitted by direct contact, and non-culturable in *Culicoides* cells	[28, 29]
BTV-26	2010	Kuwait	Sheep	Atypical; non-pathogenic, subclinical, direct contact, and non-culturable in *Culicoides* cells	[28, 30]
BTV-27	2014	Corsica, France	Goat	Atypical; non-pathogenic, subclinical, direct contact, and non-culturable in *Culicoides* cells	[28, 31]
BTV-28	2014	Middle East	Sheep (experimentally)	Clinical; contact transmitted; Seg-2 related to BTV-4, -10, -11, -17, -20, -24; Seg-5 similar to BTV-4 (SA); Other Segs similar to BTV-26	[32, 33]
BTV-X ITL2015	2015	Sardinia, Italy	Goat	Suspected new serotype; isolation not yet successful; Seg-2 related to BTV-XJ1407 (China) and BTV-27	[35]
BTV-XJ1407	2018	China	Sheep and Goat	Suspected new serotype; successful isolation; distinct phylogenetics	[36]

BTV = Bluetongue virus, Seg-2 = Segement 2, VP = Viral protein

## LIFE CYCLE

The BTV replication cycle begins with the attachment of the viral outer capsid protein VP2 to receptors on host cell surfaces. Viral entry occurs through clathrin-mediated endocytosis [43]. Acidification within early endosomes triggers the dissociation of VP2 and fusion of VP5 with the endosomal membrane, releasing the transcriptionally active viral core into the cytoplasm [44].

Inside the cytoplasm, the viral polymerase complex initiates transcription. VP1 (RNA-dependent RNA polymerase) synthesizes positive-sense ssRNA transcripts from each of the 10 genomic dsRNA segments [45]. These mRNAs are capped by VP4, which has guanylyltransferase and transmethylase activities [46], before being exported through pores at the five-fold symmetry axis of the core. Translation of VPs occurs using host ribosomes [47].

Genome replication and assembly occur in viral inclusion bodies (VIBs), where positive-sense RNAs interact with NS2, VP1, VP4, and VP6 helicase [24]. VP1 then synthesizes complementary negative strands to form dsRNA [20]. Each genome segment is packaged with its transcription complex (VP1, VP4, and VP6) within the VP3 subcore [2]. Insertion of VP7 trimers stabilizes the structure, forming a mature inner core [48].

Outer capsid proteins VP2 and VP5 are subsequently incorporated at the periphery of VIBs, producing mature virions. These are transported through the cytoplasm along vimentin-linked microtubules [16]. Viral release occurs through two mechanisms: budding, facilitated by the viroporin NS3, or host cell lysis [49].

**Figure 2:**
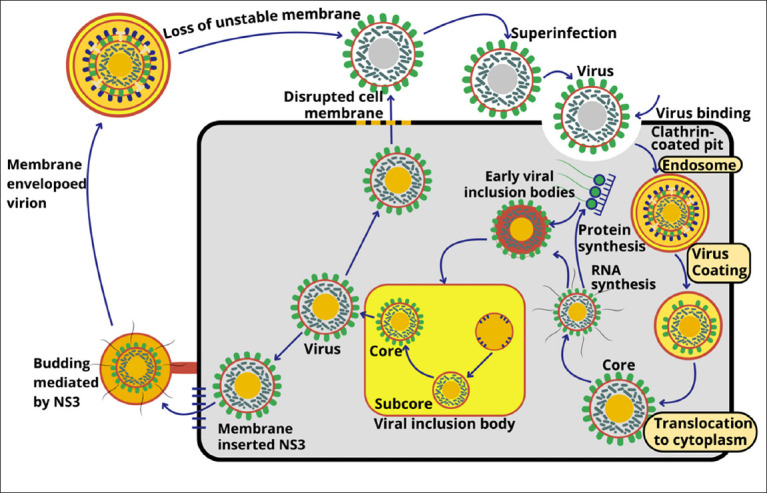
Bluetongue virus replication from entry to virion release [Source: Andi Thafida Khalisa prepared the figure].

Figure 2 illustrates the BTV replication process, from host cell entry to the release of progeny virions.

## HISTORY

The first recognized outbreak of BT occurred in the Cape Province of South Africa in the late 18^th^ century, following the introduction of fine-wooled Merino sheep from Europe [4]. Initially, the disease was described under various names, including “epizootic catarrh,” “catarrhal fever,” “malarial catarrhal fever of sheep,” and “epizootic malignant catarrhal fever of sheep,” as it was initially believed to be caused by an intraerythrocytic parasite [50]. In 1906, Theiler demonstrated that the causative agent was a filterable virus. In the same year, BTV serotype 4 was the first serotype identified in South Africa [51].

Afrikaner farmers observed cyanosis of the tongue in affected sheep and coined the term “*bloutong*” (or ”blaauwtong”), later translated into English as “*bluetongue*” by Spreull [52]. They also referred to the disease as “Bekziekte” (“mouth disease”) due to the occurrence of oral ulcers. By 1933, BT was documented in cattle, with a clinical presentation closely resembling foot-and-mouth disease, leading to confusion and alternate names such as “pseudo-foot-and-mouth disease” and “*seerbek*” [53].

Until the 1940s, BT was considered restricted to South Africa [54]. The first suspected presence outside Africa was in Cyprus in 1924, with confirmation in 1943 when BTV serotype 3 was isolated from sheep [55]. Serotype 4 was later identified in Cyprus in 1969. In 1948, the United States reported its first case, followed by the isolation of BTV-10, the first serotype described in the country [56, 57]. Subsequent serotypes included BTV-11 in 1955, BTV-17 in 1962, and BTV-13 in 1967 [58]. A severe outbreak in the Iberian Peninsula (1956–1957) involving BTV-10 caused the death of ~179,000 sheep, with mortality rates reaching 75% [59].

From Africa, BT expanded into the Middle East, Europe, North America, and Asia [55, 60]. In Europe, the first confirmed cases were reported in Greece in 1998, followed by outbreaks across the Balkans and Turkey in 1999 [61]. Between 2000 and 2002, infections spread widely across Mediterranean countries, including Italy, Spain, and Croatia [62]. In Northern Europe, BTV was first identified in the Netherlands in August 2006, and soon after, in Germany [63, 64]. In North America, BTV-2 was first detected in Florida in 1982, while BTV-1 and BTV-12 were subsequently reported in deer and cattle in Louisiana and Texas, respectively [65, 66].

In Asia, Pakistan documented BTV in 1959, isolating serotype 16 [67], followed by India’s first outbreak in Maharashtra in 1964 [68]. Malaysia provided the first serological evidence in 1977 [69], China reported isolation in Yunnan in 1979 [70], and Indonesia documented cases in imported Suffolk sheep in 1981 [71]. More recently, BTV-26 was isolated in Kuwait (2010) [72]. In Oceania, BTV was first reported in Australia in 1975, with serotype 5 identified in 2015 [60, 73]. South America reported its first cases in Brazil in 1978 [74].

## EPIDEMIOLOGY

BT is a major transboundary disease with significant socioeconomic consequences, particularly due to its impact on international trade in animals and animal products [10]. Historically, BT was considered endemic between latitudes 40°N and 35°S. However, this distribution has expanded far beyond these limits [27]. The existence of multiple serotypes and complex serological cross-relationships, ranging from partial to absent cross-protection, further complicates control efforts [4].

BTV is enzootic in ruminants and *Culicoides* vectors across tropical and temperate regions [75]. Since 1998, dramatic geographic shifts in distribution have been observed, especially in Europe [76]. New serotypes emerged in the southeastern United States, likely originating from Caribbean reservoirs, while novel introductions occurred in the Middle East and Australia [1]. Climate change has been strongly associated with the expansion of *Culicoides* vectors, particularly in Europe [77].

Currently, 28 serotypes are circulating worldwide [78]. In southern Africa, 22 endemic serotypes are co-circulating [79]. Since 1998, Europe has reported incursions of serotypes 1, 2, 4, 6, 8, 9, 11, and 16 [80]. In North America, BTV-10, 11, 13, and 17 are established [81], while additional serotypes (1, 3, 5, 6, 9, 12, 14, 19, 22, and 24) have been detected in the southeastern U.S. [81]. Between 1998 and 2005, outbreaks in 12 European countries caused over one million sheep deaths due to infection and culling [77].

Australia has reported 10 serotypes (1, 2, 3, 7, 9, 15, 16, 20, 21, and 23) [82]. In 2008, Belgium identified BTV-11, while the Netherlands and Germany reported BTV-6 [83]. Switzerland confirmed BTV-25 in 2008 [29], Kuwait detected BTV-26 in 2010 [84], and India has recorded 22 distinct serotypes [85]. In Turkey, serological surveys revealed a 90% prevalence in cattle, with serotypes 4, 9, and 16 circulating [86].

More recent outbreaks highlight the virus’s continuing expansion. In 2020–2021, BTV-1, 4, 8, 10, and 16 were reported in Oman [87], while Spain detected a novel strain of BTV-4 in the Balearic Islands in 2021 [88]. Tunisia reported BTV-8 in the same year [89], and China documented a putative novel serotype, BTV-29, in Xinjiang goats [90]. As of 2022, serotypes 3, 6, 10, 11, 12, 13, and 17 were circulating in the U.S. [91, 92]. In October 2023, Germany reported BTV-3, followed by the U.K. in March 2024 [93, 94].

By early 2025, 201 cases of BTV-3 had been confirmed in Great Britain, alongside the first-ever detection of BTV-12 in the U.K. [95, 96]. The Netherlands also confirmed BTV-12 in 2024 [97]. Despite emergency vaccination campaigns, BTV-3 resurged, spreading to Belgium, Germany, Luxembourg, France, and Denmark, resulting in widespread losses. In the Netherlands alone, ~3,807 cases were recorded, with daily sheep mortalities estimated at 1,500–1,800 and ~100,000 total deaths reported in 2024 [98]. Table 2 summarizes the reported distribution of BTV serotypes across different geographical regions from 1998 to 2025 [29-31, 76, 78, 79, 81, 82, 85-92, 94-97, 99-101]

**Table 2:** Global distribution and serotypes of BTV.

Region/Country	BTV serotypes detected	Year(s)	Notes/Remarks	References
Switzerland	25	2008	Atypical serotype; isolated from goats without clinical signs	[29]
Middle East (Oman and Kuwait)	4, 10, 16, 26	2010–2021	BTV-26 first isolated in Kuwait; BTV-4, 10, 16 detected in Oman (2020–2021)	[30, 87]
Corsica and France	27	2014	Atypical serotype; isolated from goats without clinical signs	[31]
Europe	1, 2, 4, 6, 8, 9, 11, 14, 25, 27	1998–2025	Multiple outbreaks; significant spread in northern Europe; recent novel BTV-4 strain in Balearic Islands (2021)	[76, 78, 88, 100]
Southern Africa	22 endemic serotypes	-	Co-circulation in ruminants and vectors	[79]
North America (USA)	1, 3, 5, 6, 9, 10, 11, 12, 13, 14, 16, 17, 19, 22, 24	1998–2022	New serotypes introduced from Caribbean ecosystems; multiple co-circulating strains	[81, 91, 92]
Australia	1, 2, 3, 7, 9, 15, 16, 20, 21, 23	-	Multiple endemic serotypes	[82]
India	22 distinct serotypes	-	Endemic; co-circulation	[85]
Turkey (Anatolia)	4, 9, 16	2003	90% seroprevalence in cattle; local live-attenuated vaccination for BTV-4	[86, 99]
Balearic Islands, Spain	4	2021	Novel BTV-4 strain causing outbreaks in sheep, goats, and cattle	[88]
Tunisia	8	2021	New detection of BTV-8	[89]
Xinjiang and China	29	2021	Putative novel serotype; isolated from goats	[90]
Great Britain (England and Wales)	3, 12	2024–2025	First detection of BTV-3 and BTV-12; total BTV-3 cases 201	[94, 95, 96]
Netherlands	3, 12	2024	BTV-3 outbreak despite vaccination; BTV-12 reported	[97]
North Macedonia	4	2020	First detection of BTV-4 in sheep	[101]

BTV = Bluetongue virus

Advances in genomic surveillance have greatly enhanced understanding of BTV’s evolution and epidemiology. Whole-genome sequencing has revealed that segment reassortment and point mutations drive emergence of new strains with altered virulence, host ranges, and vector competence [1]. Public repositories, such as GenBank and GISAID-Orbivirus, now facilitate real-time sequence sharing, enabling the phylogeographic tracking of outbreaks [40]. Bioinformatics tools, including codon usage bias analysis, molecular clock modeling, and antigenic cartography, further aid in assessing viral adaptation and guiding vaccine updates. Strengthening global genomic surveillance remains critical for early warning, preparedness, and coordinated responses to BT.

## PATHOGENESIS

The virus enters the host through the bite of an infected *Culicoides* midge and is transported by dendritic cells from the skin to local lymph nodes, where replication begins [5]. The virus then enters the bloodstream, causing primary viremia that disseminates to secondary organs such as the lungs, spleen, and lymph nodes [1]. BTV replicates in lymphocytes, macrophages, and vascular endothelial cells [33]. In early viremia, the virus binds to all blood components, while in late viremia, binding occurs predominantly to erythrocytes [55]. Viral particles appear to be sequestered within erythrocyte membrane invaginations, allowing prolonged viremia even in the presence of neutralizing antibodies. Free virus is present in plasma at low titers only during the early phase of infection [102].

Beyond inducing cell necrosis and apoptosis, BTV promotes vascular permeability through p38 MAP kinase activation [103]. Infection also elevates plasma concentrations of prostacyclin and thromboxane and induces the production of tumor necrosis factor-alpha, interleukin-1, interleukin-6, interleukin-8, IFN-I, and cyclooxygenase-2 [104]. These mediators often trigger excessive inflammatory responses, leading to cellular and tissue damage. BT pathology is characterized by injury to small blood vessels in target tissues, resulting in vascular occlusion and tissue infarction [54]. Infected platelets, macrophages, dendritic cells, and endothelial cells release vasoactive mediators that exacerbate endothelial injury, impair vascular function, and increase permeability, culminating in edema and effusion [105].

## IMMUNE RESPONSE

Infected animals experience a prolonged but non-persistent viremia [6]. Viral persistence depends on the lifespan of infected erythrocytes rather than other blood cells, even in late stages of infection [54]. The course of infection varies with animal species and breed: in sheep, viremia lasts 14–54 days, and in goats 19–54 days [106], while in cattle, it may persist for 60–100 days, making them key reservoirs in epidemiology [107].

BTV infection elicits both humoral and cellular immune responses, as well as interferon production [108]. Neutralizing antibodies targeting VP2 confer serotype-specific protection against reinfection with homologous strains [109]. VP5 also induces neutralizing antibodies, though at lower levels [110]. In addition, VP7 triggers the production of group-specific antibodies detectable in ruminant sera [7]. Infected animals also generate antibodies to other structural and non-structural proteins. Cell-mediated immunity begins immediately after infection and helps slow viral spread, though it cannot eliminate the virus completely [101]. CD8+ T lymphocytes play a particularly important role by exerting cytotoxic effects on infected cells [111].

## PATHOLOGY

Postmortem examinations reveal multiple gross lesions. Common findings include pulmonary edema, pleural and pericardial effusion, edema of abdominal and skeletal muscles, necrosis of myocardial and skeletal muscle, necrosis and hemorrhage of the gastrointestinal mucosa, and generalized subcutaneous, lymph node, and pregastric edema and hemorrhage [112]. Cyanosis of the tongue and oral mucosa, necrosis and flaccidity of esophageal muscles, and large volumes of frothy fluid in the trachea and bronchi is also observed [54].

Microscopic changes include macrophage infiltration around necrotic muscle fibers, fibroblast proliferation, and high collagen deposition in areas of multifocal mineralization [1]. Neutrophil infiltration may be observed in the bronchi and bronchioles, while lymphocytic and eosinophilic perivascular infiltration is often seen in the lip skin [33].

## DIAGNOSIS

BTV antigens and nucleic acids are detectable in lymph nodes, bone marrow, blood, and internal organs such as the liver, lungs, and spleen [113]. Viral recovery is most successful in the early phase of infection, though BTV can also be detected in semen [114]. Fetal and placental tissues may harbor vaccine strains and some field strains, including serotypes 3, 4, and 8 [110].

A range of molecular, serological, and virological techniques are used in diagnosis. Reverse transcription polymerase chain reaction (RT-PCR) is widely used in clinical settings to detect viral RNA and determine serotype [115]. Antigen capture ELISA can detect viral antigens, but cross-reactions between serotypes and false negatives in blood samples are common [116]. Classical virus isolation techniques employ embryonated chicken eggs or mammalian and *Culicoides* cell lines, including Kumar–*Culicoides* cells [6]. While atypical serotypes are more difficult to isolate, some strains previously believed non-cultivable have been shown to replicate in mammalian or mosquito-derived cell lines [117]. Virus isolates are typically confirmed using group-specific ELISA or immunostaining, with serotyping achieved through RT-PCR, sequencing, or virus neutralization assays [118]. However, cross-reactions can complicate interpretation of neutralization results. Historically, suckling mice and sheep were also used as sensitive models; however, this practice is now largely obsolete [6].

Serological surveillance methods include agar gel immunodiffusion (AGID), virus neutralization, and ELISA [119]. AGID cannot differentiate BTV from related orbiviruses such as epizootic hemorrhagic disease virus, but a competitive monoclonal antibody-based ELISA provides this distinction [120]. Indirect ELISA and rapid immunochromatographic tests have been developed to detect antibodies in sera, milk, and other sample types [120].

The introduction of real-time RT-PCR has revolutionized BT diagnostics, offering high sensitivity and specificity and enabling rapid outbreak investigation [121]. Segment 10 (encoding NS3) is commonly targeted to detect all serotypes, while Seg-2 (VP2) is used for serotype-specific assays, crucial for vaccine selection and outbreak management [109]. Whole-genome sequencing further enriches molecular diagnostics by characterizing genetic diversity, reassortment events, and novel serotypes [72, 84]. Expanding sequence databases now support improved RT-PCR design and phylogenetic tracking of outbreaks.

Molecular detection within vector populations has become a reference standard for virus surveillance. Importantly, real-time RT-PCR can detect viral RNA from infected midges preserved under suboptimal conditions for weeks, reducing the dependence on strict cold chain systems [11]. Table 3 summarizes the principal diagnostic techniques used for the detection and characterization of BTV in animals and vectors.

**Table 3:** Diagnostic methods for BTV.

Category	Method/Technique	Target/Sample	Advantages	Limitations/Notes
Virus isolation	Cell culture (mammalian, *Culicoides*, and Kumar–*Culicoides* cells and embryonated chicken eggs	Blood and internal tissues (liver, spleen, lymph nodes, semen, and fetus)	Produces live virus for research and vaccine validation	Some atypical serotypes are difficult to culture; requires high biosafety laboratory
PCR/RT-PCR	Conventional, real-time RT-PCR, and serotype-specific RT-PCR	Blood, tissues, vector (*Culicoides*)	Rapid, sensitive, specific; allows serotyping; supports epidemiological surveillance	Requires precise primer design; cross-contamination may affect results
Gene sequencing/Whole genome sequencing	Segment 2 (VP2), Segment 10 (NS3), and whole genome	Virus isolate or RNA	Serotype identification, genetic characterization, detection of novel serotypes, and supports vaccine matching	Expensive; requires specialized facilities and bioinformatics expertise
Antigen detection	Antigen-capture ELISA and immunostaining	Blood and tissues	Can detect viral antigen; rapid results	Cross-reactivity between serotypes; high risk of false negatives
Serology/Antibodies	AGID, VNT, ELISA (indirect, competitive), and rapid immunochromatography	Serum and bulk milk	Useful for surveillance; detects antibodies; suitable for mass screening	AGID cannot distinguish specific serotypes; risk of cross-reaction
*In vivo*	Suckling mice and experimental sheep	Virus isolate	Historically used for validation	Rarely used today due to ethical concerns and low efficiency

BTV = Bluetongue virus, RT-PCR = Reverse transcription polymerase chain reaction, ELISA = Enzyme-linked immunosorbent assay, AGID = Agar Gel Immunodiffusion, VNT = Virus neutralization test, VP = Viral protein

Field-deployable diagnostic platforms are emerging as valuable tools in resource-limited regions. Loop-mediated isothermal amplification (LAMP) and clustered regularly interspaced short palindromic repeats (CRISPR)-based assays have demonstrated excellent sensitivity and specificity, enabling near real-time farm-level testing [122]. Alongside laboratory diagnostics, digital epidemiology, using GIS, remote sensing, and livestock movement data, supports mapping of *Culicoides* hotspots, forecasting seasonal risk, and predicting high-risk trade networks [123, 124]. The integration of portable diagnostics, genomic tools, and spatial modeling forms a proactive surveillance system aligned with One Health principles.

## DIFFERENTIAL DIAGNOSIS

The clinical signs of BT can easily be confused with those of several other ruminant diseases. These include acute photosensitization, *Oestrus* *ovis* infestation, pododermatitis, acute hemonchosis (with depression and submandibular edema), sheep pox, foot-and-mouth disease, plant poisoning, facial eczema, peste des petits ruminants, pneumonia, malignant catarrhal fever, Orf (contagious pustular dermatitis), salmonellosis, rinderpest, and epizootic hemorrhagic disease [125].

## CLINICAL SYMPTOMS

In sheep, BT may present in acute, chronic, or subclinical forms, with fine-wooled breeds being especially susceptible [126]. After an incubation period of 4–8 days, clinical signs appear, including fever, lethargy, tachypnea, and hyperemia of the lips and nostrils. Excessive salivation and a serous nasal discharge initially occur, which later becomes mucopurulent and, on drying, forms crusts around the nostrils [114]. Lesions include oral mucosal ulcers, conjunctival petechiae, and edema of the tongue, lips, and submandibular region, and in some cases, edema of the ears [127]. Cyanosis of the tongue may occasionally occur. Complications such as aspiration pneumonia from regurgitation or vomiting, severe dyspnea, and hemorrhagic diarrhea are sometimes observed [54]. During the later febrile phase, sheep may develop coronitis, laminitis, or paresis. Although necrosis of striated muscles is not externally visible, it manifests clinically as reluctance to move and an arched back posture [1]. Other manifestations include dermatitis, alopecia, and torticollis. Pregnant ewes may abort, deliver mummified fetuses, or give birth to weak lambs with congenital malformations such as retinal dysplasia, brain cysts, or hydrocephalus [128]. Chronic cases often predispose sheep to bacterial pneumonia and secondary infections [129].

Goats generally exhibit subclinical infections, with overt symptoms being rare and less severe than in sheep. When present, clinical signs resemble those of sheep. During the 2006 Dutch outbreak, affected goats showed a marked reduction in milk yield, fever, edema of the head and lips, nasal discharge with scabs, erythema of the udder skin, and mild subcutaneous hemorrhagic lesions [130].

In cattle, clinical disease is uncommon, though the BTV-8 outbreak in Europe resulted in numerous symptomatic cases [107]. Clinical infection is thought to be triggered by immunoglobulin E-mediated hypersensitivity [131]. The early phase is marked by fever, apathy, and depression, followed by nasal discharge, sweating, conjunctivitis, lameness, stiffness, excessive salivation, ulcerative dermatitis, edema, hyperemia, coronitis, and occasionally bloody diarrhea [101]. Teat skin may become inflamed, cracked, and peeling, while dairy cattle show decreased milk yield [132]. Infection during early pregnancy may lead to embryo resorption, abortion, or the birth of weak or malformed calves [133]. Fetuses infected between 70 and 130 days of gestation often develop severe central nervous system abnormalities, including hydrocephalus and cerebral malformations, whereas late-gestation infection may result in mild encephalitis [134].

In white-tailed deer, acute BT closely resembles epizootic hemorrhagic disease, presenting with hemorrhagic diathesis due to disseminated intravascular coagulation [107]. Symptoms include epistaxis, bloody diarrhea, hypersalivation, edema of the head and neck, and widespread hemorrhages [135].

## TRANSMISSION

**Figure 3:**
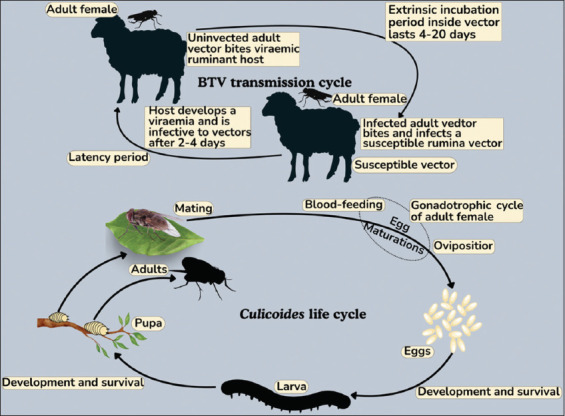
Transmission of the bluetongue virus through *Culicoides* vectors [Source: The figure was prepared by Andi Thafida Khalisa].

BT is more prevalent in lowland grasslands and valleys compared to high-altitude areas and is particularly common during late summer following rainy seasons [136]. The disease is primarily transmitted by *Culicoides* midges, small hematophagous insects now recognized as the principal vectors of BTV [83]. Experimental evidence confirms transmission: Susceptible sheep became infected after being bitten by *Culicoides* *pallidipennis* (syn. *Culicoides imicola*) that had previously fed on viremic sheep, or after inoculation with homogenates of wild-caught *Culicoides* [4]. Colonies of *Culicoides* *variipennis* have also demonstrated efficient biological transmission of BTV [137]. Figure 3 illustrates the role of *Culicoides* vectors in viral transmission.

Since their identification as BTV vectors in 1944, *Culicoides* midges have been recognized as major transmitters of BTV and other orbiviruses. Although more than 1,000 species of *Culicoides* exist globally, only a subset are efficient vectors [138]. *Culicoides* *bolitinos*, for instance, transmits BTV-1 more effectively than *C. imicola* [139]. In India, *Culicoides* *schultzei* predominates in the Marathwada and Kolkata regions, while *C. imicola* and *Culicoides* *peregrinus* dominate in Tamil Nadu [140]. In Andhra Pradesh, species such as *Culicoides* *oxystoma*, *Culicoides* majorinum, *Culicoides* *noxius*, *Culicoides* *anophelis*, *Culicoides* *peregrinus*, and *Culicoides* *actoni* have been implicated as important vectors [141].

Vector abundance is strongly seasonal, with *Culicoides* populations peaking during rainy periods, thereby driving outbreak frequency [142]. Climate change has expanded vector ranges into previously unaffected areas, resulting in outbreaks in Greece, Italy, and other BT-free regions [143]. Although mechanical transmission through sheep feces, fleas, or contaminated needles may occur, these are considered minor routes [144, 145]. Sexual transmission from infected bulls is possible, and vertical transmission through semen and across the placenta has also been documented [114].

Vector competence varies widely among *Culicoides* species, shaping regional transmission dynamics. For example, *C. imicola* is the dominant vector in Mediterranean and African regions, while *C. bolitinos* plays a greater role in cooler, high-altitude environments [146]. Climate change modeling predicts a northward expansion of competent species into temperate regions of Europe and Asia, facilitated by warming temperatures and longer activity seasons [147]. In addition to climate drivers, anthropogenic factors, such as irrigation projects, intensive livestock farming, and land-use changes, create favorable breeding habitats and boost vector abundance [147]. These findings underscore the importance of integrating entomological surveillance with climate and land-use modeling to improve outbreak prediction and support adaptive control strategies.

## ECONOMIC IMPACT

Globally, BT is responsible for estimated annual economic losses of approximately $3 billion [148]. Losses arise from both direct and indirect factors. Indirect costs include restrictions on the export of live animals, semen, and calf serum, as well as expenses related to control measures such as vaccination, surveillance, vector control, and animal movement restrictions. Direct losses encompass mortality, abortions, reduced weight gain, and decreased milk and meat production [10].

For example, the 2006–2008 BTV-8 outbreaks in northern Europe caused losses exceeding €1 billion, driven by high mortality, production declines, and severe trade restrictions [1]. More recently, the 2024 outbreak in the Netherlands resulted in the death of over 100,000 sheep, highlighting the catastrophic scale of national-level economic disruption [98].

Cost–benefit analyses consistently demonstrate that vaccination campaigns, although resource-intensive, are economically more favorable than prolonged trade bans and movement restrictions, which can devastate export markets [76]. Long-term economic modeling also shows that recurrent outbreaks erode the sustainability of wool, dairy, and meat industries, particularly in rural regions where small ruminants underpin livelihoods [10]. Hence, preventive strategies such as vaccination, vector surveillance, and international coordination are not only veterinary necessities but also economic imperatives.

In young sheep, mortality rates can range from 30% to 70%, leading to substantial productivity losses [4]. These include reproductive failures, wool shedding, and increased mortality rates during prolonged mating periods. Indirect losses, such as decreased body weight, reduced milk yield, and impaired fertility, often surpass the economic burden of non-infectious diseases [12]. International trade is also significantly impacted: Cattle and related plasma products from BTV-endemic nations face export restrictions unless animals are certified virus-free by direct detection (e.g., virus isolation or PCR) or proven seronegative by antibody testing [149]. Endemic regions therefore face ongoing financial penalties.

The lack of cross-protection among the many BTV serotypes complicates vaccination and control strategies [150, 151]. Because BT affects multiple ruminant species and is globally distributed, it remains a major concern for the World Organization for Animal Health (WOAH) and national veterinary authorities [152]. Historically, control measures have relied on clinical and serological surveillance combined with vaccination of exposed flocks [153]. BT was once listed in the WOAH “List A” of priority diseases. According to WOAH Terrestrial Code standards, all international movements of susceptible species and their potentially infectious products from infected countries are strictly prohibited until those areas are certified infection-free following a protected, vector-free period [3]. However, movement is permitted from infected countries to regions where competent *Culicoides* vectors are absent [154].

BT is a non-contagious hemorrhagic disease that affects domestic and wild ruminants, as well as camels, but poses no zoonotic risk [1]. Despite progress in developing immune-prophylactic agents, vaccines, and a wide range of diagnostic tools for rapid and reliable detection of BTV serotypes, the disease persists endemically in many regions, causing severe and recurrent economic losses [37].

## TREATMENT AND PREVENTION

There is no specific antiviral treatment for BT. Supportive care remains the cornerstone of management. Prophylactic antibiotic therapy is often administered to prevent secondary infections in symptomatic animals [6]. Supportive interventions include careful handling, provision of shelter, and the use of non-steroidal anti-inflammatory drugs when needed [11]. Clinical improvement has been documented in infected sheep treated with sodium dipyrone, penicillin, and dexamethasone acetate [155].

Prevention is primarily based on prophylactic vaccination and vector control, both of which are widely recognized as effective strategies for reducing the burden of BT [156].

### Vaccination

Vaccination can stop the spread of clinical BTor at least slow its progression by disrupting the BTV transmission cycle. This reduces the financial burden of animal infections and allows for the safe trade and movement of animals from BTV-enzootic regions [6]. Because vaccines are serotype-specific, the circulating serotypes in a region must be considered before vaccination is implemented [156]. Two main categories of vaccines are currently used to limit the spread of BT: Live attenuated and inactivated vaccines. Table 4 provides an overview of the main vaccine types used or under development for BTV control in ruminants.

**Table 4:** Overview of BTV vaccines.

Vaccine type	Key features	Advantages	Limitations/Risks	Examples/Notes
Live attenuated vaccine	Contains weakened live virus; multivalent formulations available in endemic areas	Single dose provides protection for ≥ 1 year; low production cost	May cause fetal abnormalities if given to pregnant ewes; risk of transient viremia; possible reversion to virulence or reassortment; less effective against heterologous serotypes; temperature sensitive (>35°C)	Widely used in South Africa; multivalent formulations targeting prevalent serotypes
Inactivated vaccine	Killed virus; requires revaccination	Safe, effective against homologous serotypes; prevents clinical disease; allows safe animal movement	High production cost; does not differentiate infected vs. vaccinated animals (unless DIVA applied); limited serotype coverage	Monovalent: BTV-1, -2, -4, -8, -9; Bivalent: BTV-2 and BTV-4
New-generation/experimental vaccines	Subunit, recombinant vector, virus-like particles, and DNA vaccines	Rapid immune response; no risk of viral transmission; polyvalent potential; DIVA-compatible	Higher cost; limited serotype combinations; most remain in laboratory or field trial stage	Under development; aim to improve efficacy and safety while allowing serological differentiation

BTV = Bluetongue virus, DIVA = Differentiating infected from vaccinated animals

#### Live attenuated vaccines

Live attenuated vaccines (LAVs) remain the only widely available commercial vaccines and have historically been used in endemic regions with multiple circulating serotypes, such as South Africa [157]. These areas still rely on multivalent LAVs to protect against prevalent strains [158]. Advantages include single-dose administration, at least 1 year of protection, and low production costs. However, LAVs may be less effective against heterologous serotypes and lose efficacy at high ambient temperatures (>35°C) [159].

Concerns have emerged regarding LAV use, particularly their potential to cause fetal abnormalities if ewes are vaccinated during pregnancy. Such effects may include clinical BT signs, abortion, reduced milk yield, and temporary poor semen quality in rams [160]. To mitigate risks, ewes should be vaccinated 9–15 weeks before mating, while rams should be vaccinated after mating but at least 6 weeks before the next breeding season [4]. Vaccinated animals may develop post-vaccination viremia lasting over 2 weeks [161], and reassortment between vaccine and wild-type viruses may result in reversion to virulence or emergence of recombinant strains with novel characteristics [162].

#### Inactivated vaccines

Inactivated vaccines, when properly formulated, provide reliable immunity but require revaccination for sustained protection [99]. Although more expensive to produce, they offer an optimal balance of safety and efficacy. Benefits include reduced direct economic losses, prevention of clinical disease, safe trade of vaccinated animals, and suppression of viremia following homologous infection [163].

Following the 1998 Southern European outbreak, a bivalent inactivated vaccine was produced to protect against BTV-2 and BTV-4. Monovalent inactivated vaccines targeting BTV-1, BTV-8, and BTV-9 are now also available [156]. Theoretically, these vaccines could support the development of DIVA strategies, but no practical systems have yet been commercialized [157].

Despite being central to control, vaccination faces drawbacks: Antibodies induced by vaccines cannot easily be distinguished from those arising from natural infection, and recombinant vaccine technologies face hurdles due to structural complexity of epitopes, serotype-specific immune responses, and poor antigen stability during storage [164]. Continued research is needed to develop safer, broadly protective next-generation vaccines with DIVA capabilities to address the frequent emergence of novel BTV serotypes.

#### Novel and experimental vaccines

Several innovative vaccine platforms are under development, including subunit, recombinant vector, DNA, mRNA, and VLP vaccines. These novel approaches aim to provide faster immune responses, improved safety, DIVA compatibility, and polyvalent protection [165].

DNA and mRNA vaccines show strong potential by inducing robust humoral and cellular immunity and enabling rapid adaptation to emerging serotypes [166]. VLPs mimic viral architecture without infectious material, ensuring high immunogenicity and safety [167]. Recombinant subunit vaccines targeting proteins such as VP2 and VP5 are also being evaluated as safer alternatives [156].

Recent European and Asian experimental trials (2023–2025) have demonstrated promising immunogenicity for VLP and mRNA vaccines, though large-scale validation remains limited. Challenges include high costs per protected animal, limited serotype coverage, and stability issues in field conditions. Despite these obstacles, novel vaccine research remains essential for achieving broader, safer, and more adaptable BTV immunization strategies.

## CONTROL

Suspected BT cases must be reported in compliance with local or national regulations [6]. Veterinary authorities should be consulted regarding specific requirements. Vaccination remains the primary control tool, tailored to circulating serotypes [168]. While LAVs are generally more immunogenic than inactivated vaccines, they carry risks such as fetal abnormalities in pregnant ewes and possible vector transmission [169, 170].

Vector control is considered an important complementary measure. Strategies include penning animals from dusk until dawn, avoiding damp lowland pastures, and using insect repellents or insecticide-treated netting to reduce exposure during peak feeding periods of *Culicoides* midges (sunset and sunrise) [171]. However, many species can enter barns or feed indoors, particularly late in the season, and reactivation occurs when weather cools [172]. The vast abundance and widespread habitats of *Culicoides* make sustained vector control challenging, and the level of intervention needed to significantly reduce transmission remains unclear [59].

Other control options include sentinel animal surveillance, which allows early detection of virus circulation and timely initiation of vaccination campaigns [173]. Restricting the movement of infected or in-contact animals is crucial, especially pregnant animals that test positive [1]. Additional measures include barn disinfection, equipment sanitation, and transport biosecurity, particularly after herd depopulation or before restocking [6]. Breeding schedules may also be adjusted to minimize congenital anomalies associated with infection during pregnancy [174].

At the policy level, EU Regulation 2016/429 introduced updated frameworks for BT control in April 2021, reshaping how outbreaks are managed across Europe [91].

### Integrated control strategies

Rapid BT control requires complementary approaches [164]:

- Vector management: Housing animals indoors during peak vector activity, destroying breeding sites, applying insecticides, and using repellents or ear tags. Eco-friendly measures such as neem-based biocontrol agents and larvicides are also being explored.
- Therapeutics: Although no antivirals exist, supportive care (hydration, anti-inflammatory treatment, and stress reduction) improves outcomes.
- Surveillance: Active and passive surveillance using serology, molecular diagnostics, and sentinel animals helps detect outbreaks early.
- Genetic and modeling tools: Host resistance studies and predictive models guide risk mapping and control design.
- Vaccination: Remains the cornerstone of BT prevention, with platforms including LAVs, inactivated vaccines, VLPs, DNA, and mRNA vaccines, each with unique advantages and drawbacks.

At the global level, WOAH provides surveillance and movement guidelines, while the EU Animal Health Law establishes harmonized frameworks for managing BT within Europe [175]. However, trade restrictions and vaccination policies still vary widely among nations, resulting in inconsistent outbreak responses and disproportionate economic burdens on farmers [1]. In some countries (e.g., parts of the EU during the BTV-8 epidemic), vaccination was compulsory and state-funded, whereas in others, it is voluntary, dependent on farmer awareness and costs [176]. Addressing these policy and coordination gaps is essential to enhance outbreak response, safeguard international trade, and mitigate economic disruption.

## ONE HEALTH PERSPECTIVE AND BROADER IMPLICATIONS

Although BTV is non-zoonotic, its impacts extend well beyond animal health. Climate change influences the distribution and activity of *Culicoides* vectors, altering outbreak risk and facilitating transmission at the livestock–wildlife interface [177]. These ecological changes carry profound consequences for food security, rural livelihoods, and ecosystem stability. To mitigate the socioeconomic and ecological burden of BT, a One Health approach that integrates animal, environmental, and public health considerations is essential for designing effective surveillance, vaccination, and vector control strategies [173].

BTV shares many ecological and epidemiological features with related orbiviruses, including AHSV and EHDV [83]. All three viruses are transmitted primarily by *Culicoides* midges, with overlapping vector species in certain regions, demonstrating the importance of shared ecological drivers such as climate variability and land-use modification [83]. Comparative analysis of control programs highlights valuable lessons: AHSV outbreaks in horses have been contained in endemic regions through compulsory live-attenuated vaccination, though risks of reversion to virulence and genetic reassortment remain [178]. Conversely, vaccine development for EHDV has lagged behind BTV and AHSV due to its historically lower economic significance, though recent North American outbreaks have renewed research focus [179]. These cross-Orbivirus experiences emphasize that while BTV is not zoonotic, the ecology of vector-borne Orbiviruses provides broader insights for surveillance, vaccination, and preparedness under a One Health paradigm.

The epidemiology of BTV illustrates the necessity of this framework. The virus persists at the livestock–wildlife interface, where wild ruminants may act as reservoirs that sustain circulation and facilitate spillover [180]. Vector ecology, especially the distribution and seasonality of *Culicoides* midges, is being reshaped by climatic shifts [147]. This not only heightens the risk of BTV transmission but also parallels the emergence of other vector-borne Orbiviruses with zoonotic potential. Outbreaks of BTV reduce livestock productivity, constrain international trade, and undermine rural economies dependent on small ruminant farming [181]. Although BTV does not directly infect humans, its climate-driven expansion highlights how environmental change can alter disease dynamics in ways that threaten animal health, economic stability, and, indirectly, human well-being [154].

## CONCLUSION

This review synthesizes current knowledge on BTV, highlighting its global epidemiology, molecular biology, clinical impact, diagnostics, vaccination, and control strategies. BTV remains a major transboundary disease of ruminants, with profound economic and trade implications. Its transmission is strongly linked to the ecology of *Culicoides* vectors, which are increasingly influenced by climate change, resulting in altered geographic distribution, prolonged vector activity, and expanded disease risk zones. The virus’s genetic diversity, high mutation rate, and frequent reassortment events further complicate prevention and control, while the emergence of atypical and novel serotypes underscores the need for continuous surveillance.

The strength of this review lies in its integrative approach, bringing together epidemiological trends, advances in molecular diagnostics, comparative vaccine strategies, and the role of climate and land-use changes within a One Health framework. It draws cross-Orbivirus lessons from African horse sickness and epizootic hemorrhagic disease, demonstrating how shared ecological and immunological features can inform preparedness strategies for BTV and related pathogens. From a practical perspective, vaccination remains the cornerstone of BTV prevention, with live attenuated vaccines offering cost-effective protection in endemic regions and inactivated vaccines providing safer alternatives for outbreak control and trade compliance. Emerging platforms such as VLPs, recombinant subunits, DNA, and mRNA vaccines hold promise for broader, serotype-spanning protection with potential DIVA (Differentiating Infected from Vaccinated Animals) compatibility. In addition, molecular and field-deployable diagnostic tools, including RT-PCR, LAMP, and CRISPR-based assays, are strengthening surveillance and outbreak response capacity. Vector monitoring and predictive modeling further enhance early warning systems, enabling more proactive interventions.

Despite these advances, challenges persist. Current vaccines lack broad cross-protection and are limited by serotype specificity, high production costs, and, in some cases, the risk of reversion to virulence. Vector control remains difficult due to the vast numbers and diverse habitats of *Culicoides* species. Surveillance is unevenly distributed, with resource-limited regions facing diagnostic and reporting gaps that hinder the timely detection of outbreaks. Moreover, international policy and trade restrictions remain inconsistent across regions, leading to fragmented responses.

Future research should prioritize the development of safe, cost-effective, multivalent, or pan-serotype vaccines with DIVA capabilities. Expanding whole-genome sequencing and genomic surveillance will be critical for tracking viral evolution, detecting novel serotypes, and guiding vaccine matching. Climate and land-use modeling should be integrated with vector ecology studies to refine risk prediction. A stronger emphasis on One Health approaches, incorporating animal, environmental, and socio-economic dimensions, will be vital for sustainable control. In addition, harmonized international policies and coordinated trade frameworks are needed to minimize economic disruption and enable more effective global outbreak management.

While BTV does not pose a direct zoonotic risk, its expanding distribution, driven by climate and ecological change, makes it a persistent global challenge for livestock health and trade. Strengthening surveillance, advancing vaccine innovation, improving vector management, and aligning control policies under a One Health framework are critical steps toward reducing the burden of this disease. Proactive investment in these strategies will not only safeguard ruminant health and rural livelihoods but also enhance resilience against future vector-borne viral threats.

## AUTHORS’ CONTRIBUTIONS

SRA, BWKW, WW, and ARK: Drafted the manuscript. ML, RZA, EJK, WW, and MAA: Revised and edited the manuscript. SHW, RZA, ATK, SRA, and SS: Drafted and critically revised the manuscript. RZA, WW, ARK, and IBM: Edited the references. All authors have read and approved the final version of the manuscript.
